# The Effects of Passive Simulated Jogging on Short-Term Heart Rate Variability in a Heterogeneous Group of Human Subjects

**DOI:** 10.1155/2018/4340925

**Published:** 2018-10-01

**Authors:** Jose A. Adams, Shivam Patel, Jose R. Lopez, Marvin A. Sackner

**Affiliations:** ^1^Chief, Division of Neonatology, Mount Sinai Medical Center, Miami Beach, Florida, USA; ^2^Student, University of Miami, Coral Gables, Florida, USA; ^3^Division of Neonatology, Research Scientist, USA; ^4^Emeritus Director of Medical Services, Mount Sinai Medical Center, Miami Beach, Florida, USA

## Abstract

**Background:**

Heart rate variability (HRV) reflects neural balance between sympathetic and parasympathetic autonomic nervous systems (ANS). Reduced HRV occurs in several chronic diseases and physical inactivity. External addition of pulses to the circulation restores HRV. A new method to add pulses to the circulation can be accomplished with a passive simulated jogging device (JD). We hypothesized that application of JD might increase HRV in seated and supine postures in a heterogeneous group of volunteer subjects.

**Methods:**

Twenty ambulatory persons (age range 31-88) were recruited. The physical activity intervention (JD) moved the feet in a repetitive and alternating manner; upward movement of the pedal is followed by a downward movement of the forefoot tapping against a semirigid bumper to simulate tapping of feet against the ground during jogging. Each subject underwent four, 30 min sessions in seated and supine postures with the active JD and same with Sham. HRV was assessed at baseline (BL), and Recovery (REC) from analysis of an electrocardiogram. Time domain variables were computed, namely, standard deviation of all normal RR intervals (SDNN) and square root of the mean of the sum of the squares of differences between adjacent RR intervals (RMSSD). Frequency domain measures were determined using a standard Fast Fourier spectral analysis, as well as parameters of Poincaré plots.

**Results:**

Thirty minutes of JD significantly increased time domain measures and Poincaré parameters of HRV in both seated and supine postures. Frequency domain parameters showed no change. The effects of JD on HRV measures were not affected by age, gender, or posture.

**Conclusion:**

The passive simulated jogging device increased HRV in both seated and supine postures. This intervention that provided effortless physical activity is a novel method to harness the beneficial effects of increasing HRV.

## 1. Introduction

Heart rate variability (HRV) is a measure of balance within the autonomic nervous system (ANS) of the sympathetic (SNS) and parasympathetic (PSN) nervous systems. Reduced HRV is a marker of ANS dysfunction, and occurs in diabetes, coronary artery disease, heart failure, hypertension, aging and frailty. It is prognostic in that it identifies patients with increased cardiac mortality after myocardial infarction [1–4]. Prolonged sitting and sedentary lifestyle also reduces HRV [5, 6] whereas greater leisure time activities and walking produce increased HRV [5, 7]. Prolonged sitting has been termed the “sitting disease” and carries high risk to development of heart disease, hypertension and diabetes type 2 [8]. Significant amounts of physical inactivity are present in from 18-32% of the adult population [9]. Its economic burden in the United States has been estimated at $131 billion yearly [10].

Normal HRV is beneficial in the aging and frail, those with cognitive decline, diabetes, coronary artery disease, and heart failure [4, 7, 11, 12]. Various interventions modify HRV, with physical activity being the most studied intervention [4]. Despite public knowledge of the innumerable positive effects of exercise on the cardiovascular and central nervous systems, adherence and compliance with any exercise strategy is limited.

We previously showed that addition of pulses to the circulation through whole body periodic acceleration increases heart rate variability in swine postcardiac arrest [13]. Here, we report on portable passive simulated jogging device (JD) that adds pulses to the circulation through passive tapping of the forefoot on motorized pedals that rise and as they strike a semirigid bumper to simulate the feet striking the ground with locomotion. We hypothesized that JD might increase HRV in a heterogeneous group of human subjects during physical inactivity produced by uninterrupted supine or seated postures.

## 2. Materials and Methods

### 2.1. IRB Approval

This study and its informed consent forms were approved by Western Institutional Review Board (WIRB), Study Number: 11172318 and WIRB: 20170208374 (WIRB, Puyallup, WA 98374-2115) and form part of a larger study on effects of JD in various postures. The study is registered at* ClinicalTrials.gov  *NCT03426774. Subjects were recruited by word of mouth from personal contacts. The study protocol was verbally communicated to the subject and provided with the approved written informed consent. All subjects were given the opportunity to ask questions. Interested subjects executed the written informed consent.

### 2.2. Study Participants

Twenty ambulatory individuals were recruited for this investigation by word of mouth and gave their informed consent to participate. They were asked not to drink coffee on the day of their participation and asked again about coffee drinking on subsequent days of the study. All subjects received financial remuneration for their participation. BMI was computed to characterize participants as follows: BMI normal weight 18.5 to 24.9, overweight 25 to 29.9 and obese 30 or more.  Table 1 lists the characteristics of study participants. No attempts were made to recruit a homogenous group of subjects since the intended population for use of JD was diverse ages and comorbidities.

Studies were conducted in mid-morning and early afternoon. Sessions were limited to two daily for each subject. The duration of each session comprised 15 to 20 minutes for instrumentation followed by 60 minutes thereafter for each study. Four sessions were conducted for each subject: (1) seated and supine postures with passive simulated jogging device (JD) and (2) seated and supine postures as a “Sham”. The latter consisted of placing the passive simulated jogging device (JD) on the floor adjacent to the bed or chair so that only the operational noise of the device could be heard. On study day one, subjects were sequentially assigned to start with either Seated Sham or Supine Sham and follow the flow diagram on Supplemental Data Figure 1S.

### 2.3. Passive Simulated Jogging Device (JD)

**Figure 1:**
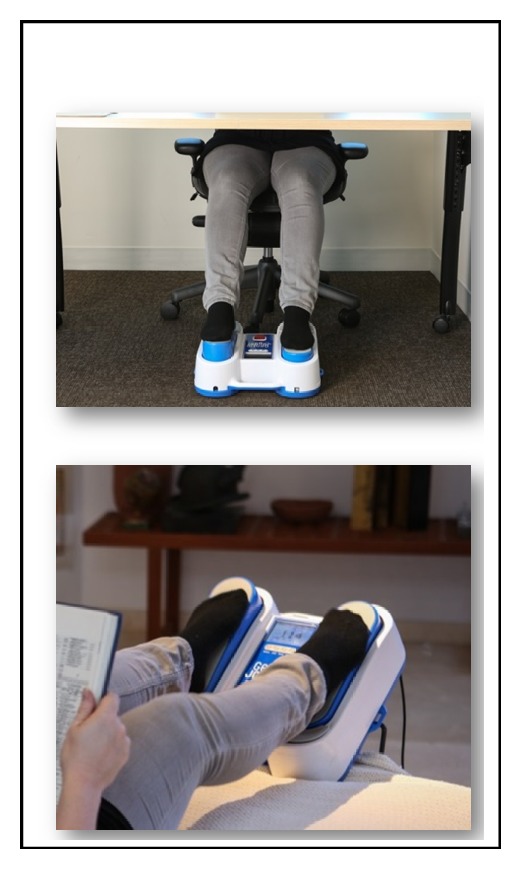
**Photographs of the passive simulated jogging device.** The photograph depicts a close-up of the feet of a seated subject upon the pedals of the passive simulated jogging device (JD). Bottom panel depicts a subject in supine posture with feet on the JD.

This portable simulated jogging device (JD) incorporates computer controlled, DC motorized movements of foot pedals placed within a chassis to repetitively tap against a semirigid surface to simulate jogging and other locomotion activities while the subject is seated or lying in a bed. It weighs about 4.5 kg with chassis dimensions of 34 x 35 x 10 cm. It is placed on the floor for seated applications and secured to the footplate of a bed for supine applications. Its foot pedals rapidly and repetitively alternate between right and left pedal movements to lift the forefeet upward about 2.54 cm followed by active downward tapping against a semirigid bumper placed within the chassis. In this manner, it simulates the impact of feet impacting against the ground [14]. Each time the foot pedals strike the bumper, a small pulse is added to the circulation. Studies were done at a cadence of (~190 steps/min).  Figure 1

### 2.4. Analysis of HRV

A three-lead electrocardiogram was used for recording of heart rate using a sampling rate of 1000 points per second (LabChart 7 PRO, ADInstruments, Colorado Springs, CO 80906).

The methods used for HRV analysis adhere to the standards developed by the Task Force of the European Society of Cardiology and the North American Society of Pacing and Electrophysiology [15]. RR intervals were obtained from the digitized electrocardiographic signals at a sample rate of 1000 points per sec. The consecutive RR intervals, which were the time intervals between successive pairs of QRS complexes where detected by using R wave detection. (LabChart7 Pro, ADInstruments, Colorado Springs, CO).

HRV was assessed by time and frequency domain methods from three 5-min consecutive RR intervals at baseline (BL), and Recovery (REC) (15 min after completion of the 30 min of JD). All ectopic beats resulting from premature ventricular contraction were removed from electrocardiographic waveforms and missing data replaced by interpolated beats derived from the nearest valid data, for analysis of normal R-R intervals, NN. The variables used for the time domain analysis include: standard deviation of all normal RR intervals (SDNN), and square root of the mean of the sum of the squares of differences between adjacent NN intervals (RMSSD). Frequency domain measures were determined using a standard Fast Fourier spectral analysis and the following calculated on the NN time intervals; very low-frequency power (VLF, 0.01–0.04 Hz), low-frequency power (LF, 0.04–0.15 Hz), and high-frequency power (HF, 0.15- 0.4Hz)., LF, and HF powers are reported in normalized units ( LFnu, and HFnu), which represent the relative value of each power component in proportion to the total power [1].

#### 2.4.1. Poincaré Measures

The Poincaré plot is based on the premise that changes in the parasympathetic and sympathetic modulation of the heart rate affect the subsequent RR intervals. Unlike frequency domain measurements Poincaré plot analysis is insensitive to changes in trends in the R-R intervals [16]. As a nonlinear method, a Poincaré plot provides a scatter of an RR interval plotted against the preceding RR interval. The analysis entails fitting an ellipse to the plot, with its center coinciding with the center of the markings. The dispersion of the points that is perpendicular to the line of identity measures the width of the Poincaré scattergram and reflects the short-term HRV. The dispersion of points along the line of identity measures the length of the scattergram and indicates the long-term HRV, which reflects the standard deviation of the RR interval (SDNN). The width (SD1) and length (SD2) of the Poincaré determine the width and length of the fitted ellipse to characterize the shape of the plot mathematically [16, 17].

### 2.5. Statistical Analysis

Analyzed EKG data were inputted into STATISTICA (StatSoft Inc., Tulsa, OK) for analysis. Mean values of each parameter along with standard deviation of the mean for each epoch. Continuous variables were evaluated by analysis of variance for repeated measures. For variables with significant differences, post hoc analysis was done using Tukey HSD for equal or unequal sample size. Comparisons of discrete variables were evaluated by Fisher's exact test. Statistical analyses were performed using STATISTICA (StatSoft Inc., Tulsa, OK). A p value of < 0.01 was considered statistically significant. Data are MEAN ± SEM.

## 3. Results

The heterogeneous study population was comprised of 20 subjects, 12 females and 8 males. The age and BMI, medications, and baseline heart rate for each subject are listed on Table 1.

### 3.1. Effects of JD in Seated and Supine Postures

**Figure 2:**
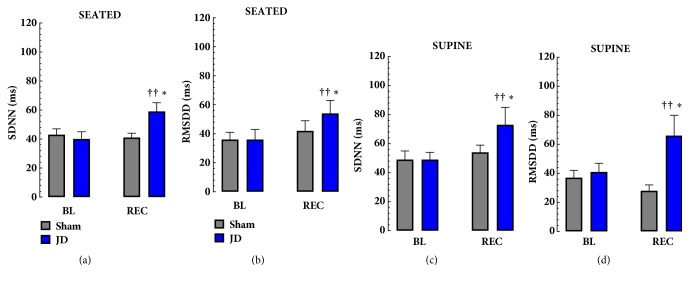
**Effects of JD on Linear Parameters of HRV**. The effects of JD on SDNN and RMSSD (linear parameters of HRV) in Seated (a, b) and Supine (c, d) Postures. JD significantly increased SDNN and RMSSD in both seated and supine postures. ††* p* < 0.05 BL vs. REC, *∗* Sham vs. JD, ^a^Seated vs. Supine (MEAN±SEM).

**Figure 3:**
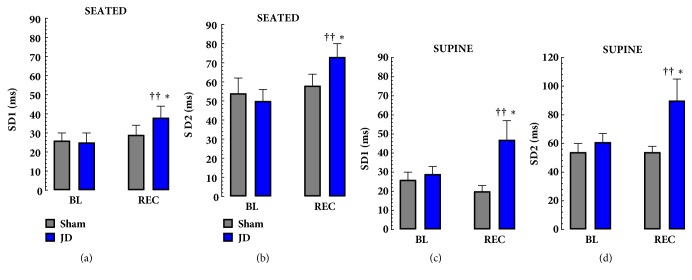
**Effects of JD on Poincaré Parameters of HRV**. The effects of JD on SD1 and SD2 (Poincaré parameters of HRV) in Seated (a,b) and Supine (c,d) postures. JD significantly increased SD1 and SD2 in both seated and supine postures. ††* p* < 0.05 BL vs. REC, *∗* Sham vs. JD, ^a^Seated vs. Supine. (MEAN±SEM).

In the both seated and supine postures 30 mins of JD increased the following measures of HRV from baseline; SDNN, RMSSD, SD1 and SD2. JD increased SDDN (Seated: BL, 40 ± 5 ms vs. JD, 59±6 ms,* p* < 0.001 and Supine: BL, 49±5 ms, vs. JD 73±12 ms,* p* < 0.001) and RMSSD (Seated: BL, 36 ± 7 ms vs. JD, 54±9 ms,* p* < 0.001 and Supine: BL, 41±6 ms, vs. JD 66±142 ms, p < 0.001). Similarly Poincaré parameters increased after JD; SD1 (Seated: BL, 25 ± 5 ms vs. JD, 38±6 ms,* p* < 0.001 and Supine: BL, 39±4 ms, vs. JD 47±10 ms,* p* < 0.01) and SD2 (Seated: BL, 50 ± 6 ms vs. JD, 73±7 ms,* p* < 0.001 and Supine: BL, 61±6 ms, vs. JD 90±15 ms,* p* < 0.01). There were no significant changes in frequency domain measures of HRV. Comparing seated and supine postures RMSDD, SD1 and SD2 were lower in supine, and SDNN was higher in supine 30 in the REC phase of JD. As expected, Sham comparisons did not show significant differences in any linear or Poincaré HRV parameters studied (see Table 2, Figures 2 and 3).

### 3.2. Effects of Gender on HRV and JD Induced Increase in HRV

Table 3 summarizes the HRV values between genders in the two postures. In the seated posture female subjects had higher heart rates compared to males in BL (Female: 73 ± 3 bpm vs. Male 62±4 bpm,* p* < 0.001) and REC (Female, 73±3 ms, vs. Male 63± 5 bpm,* p* < 0.001). In the supine posture differences in heart rate between genders were negligible. In seated posture RMSDD was also lower in female subjects at REC (Female: 49 ± 14 ms vs. Male 61±9 ms,* p* < 0.001) with similar findings in supine posture (Female: 50 ± 9 ms vs. Male 93±32 ms,* p* < 0.001). Greater SDNN, SD2, LFnu and HFnu were observed in Sham group in supine males compared to females (Table 3).

## 4. Discussion

The present study demonstrates that a single 30 min session of JD in either seated or supine postures increases short-term time domain measures of HRV and Poincaré parameters. The effects of JD on HRV measures are not age, gender, or posture dependent (see Supplemental Data File Table 1S).

In another investigation, the effects of eight minutes of passive lower limb movement on HRV were studied in the seated posture using a passive cycling device at 5-15 cycles per minute in a group of young healthy volunteers [18]. Compared to baseline values, a small decrease in linear domain parameters such as SDNN without significant change in RMSSD occurred. Additionally a decrease in Poincaré parameters such as SD2 and SD1/SD2 were observed. Decreased frequency domain parameters (total power, and low frequency) and increased high-frequency parameters were also reported. Such differences were observed mainly in young healthy male subjects. These investigators concluded that passive lower limb movement could lead to an ANS response with sympathetic nervous activity largely suppressed and vagal dominance enhanced [18]. In contrast, we found that linear parameters of HRV such as SDNN, RMSSD, and Poincaré parameters, SD1 and SD2 increased with much more rapid (~190 cpm) passive leg movements. Fujita et al. [19] who studied healthy male supine subjects using passive cycling at 60 cpm for 10 min and controlled breathing, found decreased RMSSD without changes in heart rate or frequency domain parameters. There are marked differences between their studies and ours. Shi et al. ([18] applied a frequency of passive movement to maximum of 15 cpm whereas our frequency was ~190 cpm. Time of exposure to passive movements was 8 min compared to 30 min in ours. Our study showed that both men and women respond equally to JD with increased linear and Poincaré parameters of HRV. In contrast to Fujita el al. [6], our study showed that 30 min of JD in supine posture increased both RMSSD and SDNN with no change in frequency domain parameters. We did not control respiratory rate in our study since our goal was to determine the effects of JD in real life situation on HRV parameters. Further, both Shi et al. [18] and Fujita et al. [19] studied healthy young subjects in contrast to our diversely aged population.

RMSSD is primarily reflective of parasympathetic tone whereas SDNN reflects both sympathetic and parasympathetic modulation. Low HRV values indicate a relative sympathetic dominance that may be due to high sympathetic activity and or low parasympathetic activity. Higher HRV values indicate shift of the balance towards increase parasympathetic activity [1, 16, 20]. Thus, decreased HRV values, as reported by Shi and Fujita et al. after passive exercise, indicate increase in sympathetic tone or decrease in vagal tone [18, 19].

Licurci et al. [21] studied the effects of whole body vibration (20Hz) for 10 min in older adults (mean age 64 years) in standing posture. They found significant increases in SDNN and RMSSD and none in frequency domain parameters. Our study differs with regard to JD frequency (~2.4 Hz) length of exposure to stimuli (10 min, vs. 30 min) as well as posture (standing vs. seated and supine) but our results agree that passive motion of the body increases HRV in human subjects.

Recent meta-analysis on the effects of exercise (supervised, or unsupervised and of variable intensities) increased HRV measures in individuals with various cardiovascular diseases and diabetes imply that the postexercise state is associated with increased vagal modulation and decreased sympathetic tone. These findings have also been shown by Pearson et al. [11] and others for heart failure subjects [22, 23] and those with diabetes and metabolic syndrome [12, 24, 25]. The beneficial effects of improving HRV in subjects with reduced HRV such as heart failure, diabetes, and the frail elderly has been shown by others [12, 24–26]. In heart failure subjects invasive neuromodulation strategies such as autonomic nervous system modulation using vagus nerve stimulation, renal denervation, and carotid baroreceptor stimulation are currently under investigation as a way to improve HRV [27, 28]. Since many of these patients cannot exercise due to limited exercise capacity a passive exercise strategy such as JD could be a noninvasive strategy comparable to active exercise.

The mechanisms whereby JD induces increase in HRV were not addressed in our study. Based on our previous work with whole body periodic acceleration (pGz, which also adds pulses to the circulation), we infer that the improvement of HRV with JD are in part due to increased release of nitric oxide. Nitric oxide, derived from nitric oxide synthases, neuronal nitric oxide (nNOS), and endothelial derived nitric oxide (eNOS) are important in regulating sympathetic nerve activity. Thus, pGz increases eNOS and nNOS expression in isolated cells, cardiomyocytes, and skeletal muscle [29–31]. nNOS has sympathoinhibitory effects under physiological conditions by acting on different sites of the nervous system, including paraventricular nucleus, nucleus of the solitary tract, rostral ventral medulla, the carotid body, and nerves in the kidney [32–34]. nNOS inhibition via a selective nNOS inhibitor 1-2 trifluoromethyl imidazole (TRIM) or using nNOS knockout mice markedly decreases HRV [13, 35]. In humans who received NO donor drugs or l-arginine, there has been enhanced cardiac vagal control [36].

Limitations which must be acknowledged in our study include the following. Volunteers in our study comprised a diverse group; namely, 8 had hypertension and were receiving pharmacologic therapy with 4 of them with type 2 diabetes. Moreover, they were not homogenously healthy subjects but represent a selection of real world population who might self-administer JD. Our study used JD for 30 min; long-term effects of JD on HRV require further research. Similarly to exercise continued use of JD (longer than a single session) should be undertaken in order to obtain longer lasting effects. JD introduces additional pulsations to the body by the striking of the fore foot against a bumper with each step. Thus, similarly to whole body periodic acceleration the effects are not only localized but systemic in nature, since acceleration forces can be measured at various points in the body in both supine and seated. The effects of these localized and systemic pulsations produce endothelial stimulation for production of a host of mediators including nitric oxide; in various disease states (diabetes, metabolic syndrome, limb ischemia, ischemia reperfusion injury, and others) endothelial dysfunction is not a homogenous phenomenon such that the use of JD would still be a beneficial intervention. The effects of JD on HRV in specific disease entities remain a subject for future investigation.

## 5. Conclusions

The present study demonstrates that a single 30 min session of JD in either seated or supine posture increases short-term time domain measures of HRV and Poincaré parameters in a 10-minute recovery period. The effect of short time JD usage on HRV measures were not age, gender, or posture dependent. Our findings in this heterogeneous group of human subjects suggest that increased physical activity promoted by JD can act as an intervention to improve HRV in sedentary humans.

## Figures and Tables

**Table 1:** Study Participants.

**Subject**	**Gender**	**Age (yrs)**	**BMI (kg/m** ^**2**^ **)**	**BL Seated** **HR (bpm)**	**BL Supine** **HR (bpm)**	**Medications**
1	M	55	27.8	75	75	Metoprolol/Metformin/Losartan
2	F	50	30.3	67	64	Lisinopril/Metformin/Levothyroxine
3	F	52	31.4	65	70	
4	F	41	32.3	90	77	
5	M	31	29.8	65	59	
6	F	45	20.9	69	60	
7	F	31	20.5	67	73	
8	F	38	23.2	79	69	
9	F	44	33.5	68	78	
10	F	47	35.4	73	78	
11	M	60	29.3	64	66	
12	M	68	30.7	63	55	
13	F	88	31.1	67	62	Atenolol/Amlodipine
14	M	61	24	78	78	Metoprolol
15	F	61	29.6	74	70	Lisinopril/Insulin
15	F	64	27.2	65	70	Lisinopril/Metformin
17	F	69	28.9	57	60	
18	M	68	32.1	62	62	Atenolol
19	M	63	25.8	73	72	Atenolol
20	M	60	28.2	63	59	

**MEAN**		**66.2**	**28.7**	**66.6**	**65.4**	
**SD**		**14.2**	**4.0**	**7.5**	**7.4**	

*Legend*: Characteristics of subjects enrolled in the study, including age (years), calculated body mass index (BMI (kg/m^2^)), Baseline (BL) seated, and supine heart rate (bpm=beats per min). There were 12 female subjects and 8 male subjects. Eight of the subjects were hypertensive on medications, of these 4 had type 2 diabetes mellitus.

Mean and Standard Deviation (SD).

**Table 2:** Effects of JD on HRV in seated and supine postures.

		**Seated**			**Supine**	
		**Sham**	**JD**		**Sham**	**JD**
**Heart Rate (bpm)**						
	**BL**	67(2)	69(3)		70(3)	66(2)
	**REC**	67(2)	69(3)		70(3)	65(2)
**SDNN (ms)**						
	**BL**	43(4)	40(5)		49(6)	49(5)
	**REC**	41(3)	59(6)††*∗*		54(5)	73(12) ††*∗*^a^
**RMSSD (ms)**						
	**BL**	36(5)	36(7)		37(5)	41(6)
	**REC**	42(7)	54(9) ††*∗*		28(4)^a^	66(14) ††*∗*
**SD1 (ms)**						
	**BL**	26(4)	25(5)		26(4)	29(4)
	**REC**	29(5)	38(6) ††*∗*		20(3)	47(10) ††*∗*
**SD2 (ms)**						
	**BL**	64(8)	50(6)		54(6)	61(6)
	**REC**	68(6)	73(7) ††		54(4)^a^	90(15) ††*∗*
**LF nu **						
	**BL**	55(5)	51(3)		52(6)	47(4)
	**REC**	59(6)	54(5)		57(5)	50(4)
**HF nu **						
	**BL**	35(3)	42(3)		37(4)	44(3)
	**REC**	32(4)	36(4)		34(4)	38(2)
**LF/HF**						
	**BL**	2.6(0.8)	1.7(0.4)		2.7(0.8)	1.3(0.9)*∗*
	**REC**	3.7(1.0)	2.6(0.7)		2.9(0.8)	1.5(0.2)*∗*

*Legend*: HRV parameters in seated and supine postures. Standard deviation of all normal RR intervals (SDNN), square root of the mean of the sum of the squares of differences between adjacent NN intervals (RMSSD). Poincare parameters of SD1 and SD2, frequency domain parameters determined using a standard Fast Fourier spectral analysis calculated on the NN time intervals; low-frequency power (LF) high-frequency power (HF) LF, and HF powers are reported in normalized units (LFnu, and HFnu) Baseline (BL) and Recovery (REC) in seated and supine postures for Sham and JD. ††*p* < 0.05 BL vs. REC. *∗* Sham vs. JD. ^a^Seated vs. Supine.

**Table 3:** The effects of gender on JD induced changes in HRV in seated and supine postures.

		**SEATED**				**SUPINE**			
		**Female**		**Male**		**Female**		**Male**	
		**Sham**	**JD**	**Sham**	**JD**	**Sham**	**JD**	**Sham**	**JD**
**Heart Rate (bpm)**									
	BL	75(4)	73(3)	64(4)†	62(4) †	71(3)	68(3)	62(4)†	62(3) †
	REC	73(4)	73(3)	65(4) †	63(5) †	70(3)	67(3)	64(4)	61(4)
**SDNN (ms)**									
	BL	41(6)	40(8)	46(6)	41(6)	41(4)	46(5)	61(12) †	53(10)
	REC	35(3)	59(10)*∗*††	49(5) †	59(5) ††	47(5)	62(6) *∗*††	62(9) †	92(31)†††
**RMSSD (ms)**									
	BL	32(8)	39(10)	44(5) †	31(5)	35(7)	37(6)	39(9)	48(11)
	REC	22(4)	49(14) *∗*	37(6) †	61 (9)*∗*†††	43(10)	50(9) ††	40(10)	93(32)*∗*†††
**SD1 (ms)**									
	BL	22(6)	27(7)	31(3)	22(4) *∗*	24(5)	26(4)	28(6)	34(8)
	REC	15(3)	35(10) *∗*	26(5) †	43(6) *∗*†††	30(7)	36(7)	28(7)	66(23)*∗*†††
**SD2 (ms)**									
	BL	52(8)	49(9)	57(8)	52(7)	51(3)	59(6)	82(16) †	66(12)
	REC	47(4)	75(11)*∗*††	63(8) †	70(6) ††	58(5)	78(7) *∗*††	83(12) †	110(39)†††
**LF nu **									
	BL	58(7)	51(5)	50(6)	50(4)	57(8)	46(5)	45(8)	50(8)
	REC	63(7)	61(6)	52(11)	42(8) †	48(6)	53(4)	69(7) †††	46(9) *∗*
**HF nu **									
	BL	31(5)	42(5)	39(3)	42(3)	34(6)	47(4) *∗*	42(6)	39(6)
	REC	30(5)	31(4) ††	35(7)	47(7) †	41(4)	39(2)	25(5) †††	35(5)
**LF/HF**									
	BL	3.5(1.3)	1.9(0.6)	1.4(0.3) †	1.3(0.2)	2.9(0.8)	1.2(0.2) *∗*	2.5(1.6)	1.5(0.3)
	REC	4.2(1.4)	3.1(0.8)††	3.0(1.3)	1.8(1.1) †	1.6(0.5)	1.5(0.2)	4.8(1.6) †	1.5(0.4) *∗*

*Legend*: HRV parameters in seated and supine postures for females and male subjects. Standard deviation of all normal RR intervals (SDNN), square root of the mean of the sum of the squares of differences between adjacent NN intervals (RMSSD). Poincare parameters of SD1 and SD2 and frequency domain parameters determined using a standard Fast Fourier spectral analysis calculated on the NN time intervals; low-frequency power (LF), high-frequency power (HF) LF, and HF powers are reported in normalized units (LFnu, and HFnu) Baseline (BL) and Recovery (REC) in seated and supine postures for Sham and JD. †*p*< 0.05 Female vs. Male. ††*p* < 0.05 BL vs. REC. *∗* Sham vs. JD. ^a^Seated vs. Supine.

## Data Availability

The electronic spreadsheet data used to support the findings of this study are available from the corresponding author upon request. Additionally, data used to support the findings of this study are also included within the article, as a supplemental file (Supplemental Data File Table 2S).

## Supplementary Materials

Supplementary Materials
***Table*** ***1S. *The effects of age on JD induced changes in HRV**.* Legend*: The effects of age on HRV parameters in seated and supine postures for older (subjects >59 yrs) and younger (subjects < 59 yrs). Standard deviation of all normal RR intervals (SDNN), square root of the mean of the sum of the squares of differences between adjacent NN intervals (RMSSD). Poincaré parameters of SD1 and SD2 and frequency domain parameters determined using a standard Fast Fourier spectral analysis calculated on the NN time intervals; low-frequency power (LF), high-frequency power (HF) LF, and HF powers are reported in normalized units (LFnu, and HFnu) Baseline (BL) and Recovery (REC) in seated and supine postures for Sham and JD. †* p*< 0.05 Older vs. Younger. ††* p* < 0.05 BL vs. REC, *∗* Sham vs. JD. ^a^Seated vs. Supine.***Table******2S. *Raw Data for HRV Parameters Analyzed**.* Legend*: Table of raw data including subject number, age (o=old (>59yr) y= young (59yr), type of intervention (GJ=Jogging Device, Sham), condition (BL=baseline, REC=Recovery), and HRV parameters.** Figure ****1S. Consort Flow Diagram**.* Legend*: Twenty participants were enrolled and completed the entire study. Since the study was designed to mimic real world situation, no attempts were made to exclude any subjects.
