# Supplementary material for: The Effects of Passive Simulated Jogging on Short-Term Heart Rate Variability in a Heterogeneous Group of Human Subjects
Source: J Sports Med (Hindawi Publ Corp). 2018 Oct 1;2018:4340925. doi: 10.1155/2018/4340925 (PMC6191954; doi:10.1155/2018/4340925)
Supplement: Supplementary Materials — Table 1S. The effects of age on JD induced changes in HRV. Legend: The effects of age on HRV parameters in seated and supine postures for older (subjects >59 yrs) and younger (subjects < 59 yrs). Standard deviation of all normal RR intervals (SDNN), square root of the mean of the sum of the squares of differences between adjacent NN intervals (RMSSD). Poincaré parameters of SD1 and SD2 and frequency domain parameters determined using a standard Fast Fourier spectral analysis calculated on the NN time intervals; low-frequency power (LF), high-frequency power (HF) LF, and HF powers are reported in normalized units (LFnu, and HFnu) Baseline (BL) and Recovery (REC) in seated and supine postures for Sham and JD. † p< 0.05 Older vs. Younger. †† p < 0.05 BL vs. REC, ∗ Sham vs. JD. aSeated vs. Supine.Table2S. Raw Data for HRV Parameters Analyzed. Legend: Table of raw data including subject number, age (o=old (>59yr) y= young (59yr), type of intervention (GJ=Jogging Device, Sham), condition (BL=baseline, REC=Recovery), and HRV parameters. Figure 1S. Consort Flow Diagram. Legend: Twenty participants were enrolled and completed the entire study. Since the study was designed to mimic real world situation, no attempts were made to exclude any subjects. [file 4340925.f1.pdf]

Supplementary Information
Figure 1S

CONSORT Flow Diagram

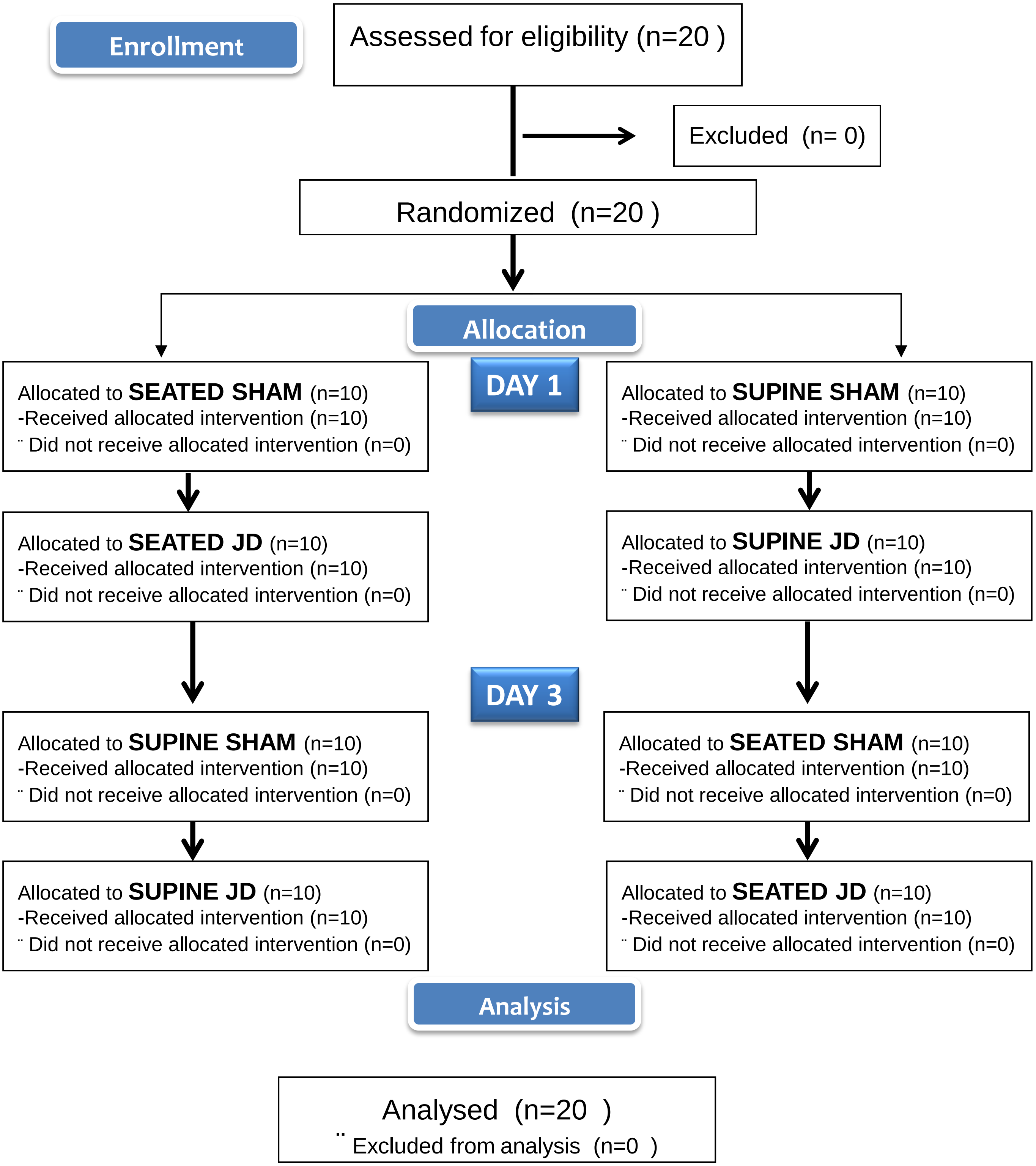

Supplemental Data

Table 1S. The effects of age on JD induced changes in HRV

		SEATED				SUPINE			
		Older		Younger		Older		Younger	
		Sham	JD	Sham	JD	Sham	JD	Sham	JD
Heart Rate (bpm)									
	BL	65(3)	67(3)	77(4) †	71(4)	62(3)	65(3)	73(3) †	67(3)
	REC	64(3)	68(4)	73(6) †	70(4)	62(3)	65(3)	74(3) †	65(3)
SDNN (ms)									
	BL	44(6)	33(6)	39(5)	48(8) †	50(6)	43(6)	49(11)	55(8)†
	REC	43(4)	51(9)* ††	48(8)	68(9)* †††	57(6)	56(6) ††	50(8)	92(25)*† ††
RMSSD (ms)									
	BL	42(7)	27(7)*	32(8) †	46(12) †	39(8)	35(7)	34(8)	49(9)†
	REC	35(6)	46(11) ††	21(4) †	63(15)* †††	44(11)	50(10) ††	39(9)	84(27)*† ††
SD1(ms)									
	BL	29(5)	19(5)*	15(3) †	32(8)* †	27(5)	25(5)	24(6)	35(6)* †
	REC	25(4)	32(8) ††	32(8) †††	44(11)* †††	31(8)	36(7)	27(6)	60(19)*† ††
SD2(ms)									
	BL	58(8)	43(7)*	54(8)	58(10) †	63(9)	54(7)	65(15)	70(9) †
	REC	54(6)	64(11) ††	53(7)	83(10)* †††	73(8)	68(8) ††	64(10)	115(30)*† ††
LF nu									
	BL	53(7)	51(4)	57(7)	50(6)	44(8)	49(6)	61(7) †	45(5)*
	REC	47(9)	56(8)	72(5) †††	52(6)*	60(8)	48(7)	52(6)	53(4)
HF nu									
	BL	37(5)	42(4)	32(4)	42(5)*	41(5)	40(4)	33(7) †	48(5)* †
	REC	39(6)	35(6)	24(5) †	39(5)*	30(5)	38(4)	39(5)	38(2)
LF/HF									
	BL	2.9(1.5)	1.4(0.4)	2.3(0.5)	1.9(0.8)	2.0(0.9)	1.5(0.3)	3.5(1.4)†	1.2(0.3)*
	REC	2.6(1.4)	2.9(0.9)	4.8(1.4)† ††	2.3(1.0)	3.3(1.1)	1.5(0.3)	2.5(1.3)	1.5(0.2)

Legend:

The effects of age on HRV parameters in seated and supine postures for Older (subjects >59 yrs) and Younger (subjects < 59 yrs). Standard deviation of all normal RR intervals (SDNN), square root of the mean of the sum of the squares of differences between adjacent NN intervals (RMSSD). Poincare parameters of SD1 and SD2, Frequency domain parameters determined using a standard Fast Fourier spectral analysis calculated on the NN time intervals; low-frequency power (LF) , high-frequency power (HF) LF, and HF powers are reported in normalized units (LFnu, and HFnu) Baseline (BL) and recovery (REC) in seated and supine postures for Sham and JD . † $p < 0.05$ Older vs. Younger †† $p < 0.05$ BL vs. REC , * Sham vs. JD, [a] Seated vs. Supine

Supplementary Information

Tabel 2 S

BASELINE TO RECOVERY					*SDNN*	*SD Delta N*	*RMSSD*	*SD1*	*SD2*	*Heart Rate*	*LF Power*	*HF Pov*	*LF/HF*
SUBJECT	*AGE*	*TYPE*								*BPM*	*nu*	*nu*	
1	o	GJ	SEATED	**BL**	49.1	41.9	41.8	29.7	62.7	43	69	31	2.2
1	o	GJ	SEATED	**REC**	52.6	43.3	43.2	30.6	67.8	44	64	36	1.8
1	o	GJ	SUPINE	**BL**	72.0	58.6	58.5	41.4	93.0	45	64	36	1.7
1	o	GJ	SUPINE	**REC**	99.5	63.8	63.7	45.1	133.3	51	72	27	2.7
1	o	SHAM	SEATED	**BL**	46.5	49.5	49.4	35.0	55.6	41	54	46	1.2
1	o	SHAM	SEATED	**REC**	57.9	43.7	43.6	30.9	75.8	40	56	44	1.3
1	o	SHAM	SUPINE	**BL**	63.7	54.9	54.8	38.8	81.2	41	60	40	1.5
1	o	SHAM	SUPINE	**REC**	88.6	53.4	53.2	37.7	119.6	44	73	27	2.7
2	y	GJ	SEATED	**BL**	25.7	19.9	19.8	14.1	33.6	58	57	40	1.4
2	y	GJ	SEATED	**REC**	37.8	25.6	25.5	18.1	50.3	60	46	45	1.0
2	y	GJ	SUPINE	**BL**	51.2	39.8	39.7	28.1	66.7	53	53	45	1.2
2	y	GJ	SUPINE	**REC**	46.8	30.6	30.5	21.6	62.6	54	54	44	1.2
2	y	SHAM	SEATED	**BL**	33.4	11.4	11.4	8.1	46.6	72	77	18	4.3
2	y	SHAM	SEATED	**REC**	26.7	12.9	12.8	9.1	36.7	72	84	11	7.7
2	y	SHAM	SUPINE	**BL**	32.9	25.4	25.4	18.0	43.0	64	78	17	4.5
2	y	SHAM	SUPINE	**REC**	49.5	67.4	67.3	47.7	51.2	66	24	48	0.5
3	Y	GJ	SEATED	**BL**	42.7	34.1	34.1	24.1	55.4	65	67	30	2.3
3	Y	GJ	SEATED	**REC**	81.8	37.6	37.6	26.6	112.6	69	69	27	2.5
3	Y	GJ	SUPINE	**BL**	35.8	23.7	23.6	16.7	47.8	67	64	31	2.0
3	Y	GJ	SUPINE	**REC**	48.7	29.5	29.5	20.9	65.6	65	71	27	2.6
3	Y	SHAM	SEATED	**BL**	54.2	66.5	66.4	47.0	60.5	70	32	39	0.8
3	Y	SHAM	SEATED	**REC**	41.5	30.6	30.5	21.6	54.6	71	66	30	2.2
3	Y	SHAM	SUPINE	**BL**	43.6	29.3	29.3	20.7	58.0	69	66	31	2.1
3	Y	SHAM	SUPINE	**REC**	38.7	21.7	21.7	15.3	52.5	74	48	49	1.0
4	o	GJ	SEATED	**BL**	37.62	29.12	29.07	20.59	49.06	55.28	58.49	40.31	1.45
4	o	GJ	SEATED	**REC**	73.11	87.02	86.86	61.53	83.09	56.97	28.44	56.90	0.50
4	o	GJ	SUPINE	**BL**	38.92	28.67	28.62	20.27	51.17	58.80	65.94	31.95	2.06
4	o	GJ	SUPINE	**REC**	40.59	50.76	50.66	35.89	44.79	48.88	34.88	64.80	0.54
4	o	SHAM	SEATED	**BL**	37.56	26.26	26.22	18.57	49.77	61.68	60.41	35.24	1.71

4 o	SHAM	SEATED	**REC**	53.64	25.24	25.20	17.85	73.73	63.73	65.87	30.13	2.19
4 o	SHAM	SUPINE	**BL**	79.32	28.84	28.79	20.39	110.31	57.86	50.43	46.41	1.09
4 o	SHAM	SUPINE	**REC**	39.03	18.71	18.68	13.23	53.59	55.98	69.80	27.78	2.51
5 y	GJ	SEATED	**BL**	44.6	36.3	36.3	25.7	57.7	86.8	46.5	42.6	1.1
5 y	GJ	SEATED	**REC**	65.5	37.1	37.0	26.2	88.8	83.7	66.6	28.4	2.3
5 y	GJ	SUPINE	**BL**	54.4	35.6	35.5	25.2	72.7	69.5	37.3	51.3	0.7
5 y	GJ	SUPINE	**REC**	76.8	46.7	46.7	33.0	103.5	66.4	55.0	39.2	1.4
5 y	SHAM	SEATED	**BL**	50.61	37.58	37.53	26.57	66.45	79.30	57.44	34.26	1.68
5 y	SHAM	SEATED	**REC**	44.46	32.35	32.31	22.87	58.56	77.70	58.02	36.76	1.58
5 y	SHAM	SUPINE	**BL**	38.04	30.87	30.83	21.83	49.17	72.85	52.05	42.05	1.24
5 y	SHAM	SUPINE	**REC**	38.59	26.67	26.64	18.86	51.21	74.57	58.54	38.23	1.53
6 o	GJ	SEATED	**BL**	35.70	39.76	39.71	28.11	41.93	76.72	33.30	34.12	0.98
6 o	GJ	SEATED	**REC**	67.19	71.98	71.89	50.90	80.24	81.96	27.42	32.62	0.84
6 o	GJ	SUPINE	**BL**	47.48	71.41	71.32	50.50	44.25	77.96	8.22	26.55	0.31
6 o	GJ	SUPINE	**REC**	69.57	107.48	107.34	76.00	62.47	76.92	8.53	31.04	0.27
6 o	SHAM	SEATED	**BL**	40.48	41.47	41.42	29.33	49.17	71.70	48.43	47.06	1.03
6 o	SHAM	SEATED	**REC**	49.96	42.60	42.54	30.12	63.91	71.36	27.65	58.26	0.47
6 o	SHAM	SUPINE	**BL**	58.21	44.56	44.49	31.51	76.05	68.50	36.74	37.41	0.98
6 o	SHAM	SUPINE	**REC**	76.84	46.48	46.41	32.87	103.58	65.21	58.80	28.15	2.09
7 y	GJ	SEATED	**BL**	11.72	10.09	10.07	7.13	14.96	88.45	44.37	42.80	1.04
7 y	GJ	SEATED	**REC**	40.39	28.88	28.84	20.42	53.35	80.51	51.90	38.82	1.34
7 y	GJ	SUPINE	**BL**	52.56	48.50	48.44	34.29	65.95	81.43	21.13	66.84	0.32
7 y	GJ	SUPINE	**REC**	57.81	51.29	51.22	36.27	73.28	73.12	42.18	46.06	0.92
7 y	SHAM	SEATED	**BL**	15.48	6.58	6.58	4.66	21.39	97.13	61.64	31.17	1.98
7 y	SHAM	SEATED	**REC**	35.87	8.34	8.33	5.90	50.39	90.50	72.67	23.81	3.05
7 y	SHAM	SUPINE	**BL**	29.71	20.71	20.68	14.64	39.38	76.50	69.91	20.87	3.35
7 y	SHAM	SUPINE	**REC**	24.83	11.50	11.48	8.13	34.16	80.00	63.19	32.23	1.96
8 y	GJ	SEATED	**BL**	45.58	42.79	42.73	30.26	56.92	68.45	16.45	77.16	0.21
8 y	GJ	SEATED	**REC**	70.97	62.04	61.95	43.87	90.27	67.88	51.05	37.20	1.37
8 y	GJ	SUPINE	**BL**	41.94	46.38	46.31	32.80	49.42	66.34	18.66	76.48	0.24
8 y	GJ	SUPINE	**REC**	71.44	45.51	45.45	32.18	95.76	71.23	55.36	38.46	1.44
8 y	SHAM	SEATED	**BL**	37.77	20.82	20.80	14.72	51.35	84.61	41.65	53.40	0.78
8 y	SHAM	SEATED	**REC**	37.59	21.14	21.12	14.95	51.02	88.56	45.37	48.88	0.93
8 y	SHAM	SUPINE	**BL**	38.31	30.44	30.40	21.52	49.72	81.66	26.69	66.47	0.40

8	y	SHAM	SUPINE	**REC**	45.11	36.80	36.76	26.02	58.24	78.71	42.61	50.47	0.84
9	o	GJ	SEATED	**BL**	20.88	16.13	16.10	11.40	27.24	61.46	47.71	50.51	0.94
9	o	GJ	SEATED	**REC**	27.34	14.25	14.22	10.07	37.33	65.40	73.40	25.08	2.93
9	o	GJ	SUPINE	**BL**	28.40	18.24	18.21	12.90	38.04	61.28	70.26	26.49	2.65
9	o	GJ	SUPINE	**REC**	36.80	17.50	17.47	12.38	50.54	60.84	62.94	35.67	1.76
9	o	SHAM	SEATED	**BL**	86.93	64.45	64.36	45.58	114.17	70.18	33.91	41.14	0.82
9	o	SHAM	SEATED	**REC**	37.96	22.97	22.93	16.24	51.16	63.11	44.49	53.18	0.84
9	o	SHAM	SUPINE	**BL**	41.02	67.81	67.70	47.95	32.65	61.75	4.80	57.40	0.08
9	o	SHAM	SUPINE	**REC**	75.99	116.78	116.58	82.58	68.78	59.24	14.39	55.56	0.26
10	o	GJ	SEATED	**BL**	12.70	5.12	5.12	3.62	17.60	81.96	72.87	22.84	3.19
10	o	GJ	SEATED	**REC**	21.43	4.80	4.80	3.40	30.11	80.97	84.68	13.50	6.27
10	o	GJ	SUPINE	**BL**	28.14	15.63	15.61	11.05	38.23	70.42	38.25	55.84	0.69
10	o	GJ	SUPINE	**REC**	36.19	11.34	11.33	8.02	50.55	75.89	57.26	39.45	1.45
10	o	SHAM	SEATED	**BL**	16.60	3.39	3.39	2.40	23.35	79.60	90.83	5.74	15.82
10	o	SHAM	SEATED	**REC**	26.94	4.23	4.23	2.99	37.98	81.13	92.15	6.48	14.22
10	o	SHAM	SUPINE	**BL**	28.39	13.85	13.83	9.80	38.94	70.95	89.82	9.41	9.54
10	o	SHAM	SUPINE	**REC**	32.17	14.26	14.24	10.08	44.37	70.31	85.30	13.51	6.31
11	o	GJ	SEATED	**BL**	12.46	5.51	5.50	3.90	17.19	82.01	68.65	26.38	2.60
11	o	GJ	SEATED	**REC**	23.77	4.25	4.24	3.00	33.49	82.11	82.74	14.65	5.65
11	o	GJ	SUPINE	**BL**	28.15	15.70	15.68	11.10	38.23	70.40	38.06	56.06	0.68
11	o	GJ	SUPINE	**REC**	37.83	13.30	13.28	9.40	52.67	76.55	68.82	28.21	2.44
11	o	SHAM	SEATED	**BL**	57.87	83.05	82.91	58.73	56.99	58.25	21.82	58.36	0.37
11	o	SHAM	SEATED	**REC**	30.75	48.33	48.25	34.18	26.88	59.20	8.97	51.84	0.17
11	o	SHAM	SUPINE	**BL**	63.99	77.21	77.08	54.60	72.17	60.12	28.98	54.26	0.53
11	o	SHAM	SUPINE	**REC**	66.68	71.08	70.96	50.26	79.79	63.50	35.72	43.10	0.83
12	y	GJ	SEATED	**BL**	75.35	56.90	56.80	40.23	98.68	59.22	49.04	43.59	1.13
12	y	GJ	SEATED	**REC**	74.78	74.39	74.24	52.60	91.75	49.79	49.26	50.59	0.97
12	y	GJ	SUPINE	**BL**	103.70	106.68	106.50	75.44	125.76	58.03	38.88	58.43	0.67
12	y	GJ	SUPINE	**REC**	308.93	311.68	311.16	220.39	377.23	59.73	54.15	25.61	2.11
12	y	SHAM	SEATED	**BL**	82.34	51.28	51.20	36.26	110.66	58.92	75.42	22.08	3.42
12	y	SHAM	SEATED	**REC**	74.14	38.02	37.97	26.88	101.35	63.91	75.18	21.70	3.46
12	y	SHAM	SUPINE	**BL**	130.86	87.07	86.90	61.57	174.52	54.47	28.73	59.76	0.48
12	y	SHAM	SUPINE	**REC**	105.78	86.50	86.35	61.17	136.52	57.48	51.54	39.30	1.31
13	o	GJ	SEATED	**BL**	26.64	17.81	17.79	12.60	35.51	69.90	53.70	39.42	1.36

13	o	GJ	SEATED	**REC**	34.36	9.92	9.91	7.02	48.08	80.39	88.08	9.28	9.49
13	o	GJ	SUPINE	**BL**	21.65	12.05	12.03	8.52	29.41	70.63	57.90	34.10	1.70
13	o	GJ	SUPINE	**REC**	58.04	65.60	65.51	46.39	67.72	72.68	44.45	35.16	1.26
13	o	SHAM	SEATED	**BL**	27.25	24.78	24.75	17.52	34.33	69.80	46.63	35.49	1.31
13	o	SHAM	SEATED	**REC**	51.89	71.27	71.17	50.40	53.35	69.29	11.73	65.98	0.18
13	o	SHAM	SUPINE	**BL**	15.58	12.17	12.16	8.61	20.28	71.70	35.72	57.51	0.62
13	o	SHAM	SUPINE	**REC**	47.26	14.76	14.74	10.44	66.01	73.15	90.31	8.00	11.29
14	Y	GJ	SEATED	**BL**	29.38	11.65	11.64	8.24	40.72	79.60	84.29	9.48	8.89
14	Y	GJ	SEATED	**REC**	32.91	11.59	11.57	8.19	45.81	82.96	90.60	8.04	11.27
14	Y	GJ	SUPINE	**BL**	18.22	12.45	12.43	8.80	24.22	78.73	64.85	22.78	2.85
14	Y	GJ	SUPINE	**REC**	55.99	77.95	77.85	55.12	56.85	77.60	25.39	39.05	0.65
14	Y	SHAM	SEATED	**BL**	23.10	9.09	9.08	6.43	32.03	88.57	78.20	15.62	5.01
14	Y	SHAM	SEATED	**REC**	24.40	9.34	9.32	6.60	33.87	84.85	88.31	7.87	11.22
14	Y	SHAM	SUPINE	**BL**	21.02	10.56	10.55	7.47	28.77	89.43	72.59	17.81	4.07
14	Y	SHAM	SUPINE	**REC**	29.68	22.58	22.56	15.97	38.82	84.32	56.34	27.49	2.05
15	o	GJ	SEATED	**BL**	31.99	23.33	23.29	16.50	42.12	59.32	37.98	57.22	0.66
15	o	GJ	SEATED	**REC**	43.69	30.58	30.53	21.63	57.87	56.03	76.19	22.82	3.34
15	o	GJ	SUPINE	**BL**	39.94	26.07	26.02	18.43	53.39	54.20	64.39	33.41	1.93
15	o	GJ	SUPINE	**REC**	54.18	34.30	34.24	24.26	72.68	52.70	63.90	33.70	1.90
15	o	SHAM	SEATED	**BL**	42.93	25.01	24.97	17.69	58.08	60.03	79.43	18.32	4.33
15	o	SHAM	SEATED	**REC**	51.23	28.59	28.54	20.22	69.57	56.60	66.18	31.23	2.12
15	o	SHAM	SUPINE	**BL**	44.12	16.04	16.02	11.34	61.36	60.13	77.19	19.60	3.94
15	o	SHAM	SUPINE	**REC**	57.42	25.18	25.13	17.80	79.24	50.91	61.09	37.43	1.63
16	o	GJ	SEATED	**BL**	24.86	8.27	8.26	5.85	34.67	71.59	51.72	46.38	1.12
16	o	GJ	SEATED	**REC**	52.42	58.22	58.14	41.17	61.65	70.86	23.42	67.25	0.35
16	o	GJ	SUPINE	**BL**	24.21	16.26	16.23	11.49	32.25	61.42	72.01	25.01	2.88
16	o	GJ	SUPINE	**REC**	40.61	24.23	24.19	17.13	54.81	63.61	70.74	26.40	2.68
16	o	SHAM	SEATED	**BL**	38.0	57.6	57.5	40.8	35.1	65.8	33.0	47.7	0.7
16	o	SHAM	SEATED	**REC**	29.6	42.4	42.3	30.0	29.1	72.1	13.0	33.2	0.4
16	o	SHAM	SUPINE	**BL**	34.7	18.4	18.4	13.0	47.3	66.7	24.7	37.7	0.7
16	o	SHAM	SUPINE	**REC**	27.4	15.1	15.0	10.6	37.3	63.9	81.7	16.9	4.8
17	o	GJ	SEATED	**BL**	34.59	26.85	26.81	18.99	45.08	62.61	36.01	57.53	0.63
17	o	GJ	SEATED	**REC**	43.01	55.77	55.68	39.43	46.32	60.33	25.66	60.72	0.42
17	o	GJ	SUPINE	**BL**	77.21	65.37	65.27	46.23	98.92	62.44	24.53	69.84	0.35

17 o	GJ	SUPINE	**REC**	64.67	83.91	83.79	59.34	69.60	67.97	14.00	39.35	0.36
17 o	SHAM	SEATED	**BL**	51.01	41.23	41.18	29.16	65.99	72.18	57.11	34.94	1.63
17 o	SHAM	SEATED	**REC**	42.56	17.66	17.63	12.49	58.87	68.38	79.90	17.61	4.54
17 o	SHAM	SUPINE	**BL**	67.95	52.69	52.61	37.26	88.58	64.96	32.71	48.06	0.68
17 o	SHAM	SUPINE	**REC**	62.10	68.34	68.24	48.32	73.33	72.22	32.89	42.28	0.78
18 y	GJ	SEATED	**BL**	39.90	30.28	30.23	21.41	52.20	56.60	52.37	44.76	1.17
18 y	GJ	SEATED	**REC**	72.30	87.05	86.90	61.56	81.64	56.91	26.06	58.76	0.44
18 y	GJ	SUPINE	**BL**	38.76	28.70	28.65	20.29	50.93	58.79	66.00	31.90	2.07
18 y	GJ	SUPINE	**REC**	54.20	37.05	36.98	26.20	72.04	50.83	66.21	33.30	1.99
18 y	SHAM	SEATED	**BL**	49.11	61.55	61.47	43.52	54.12	74.41	21.24	43.03	0.49
18 y	SHAM	SEATED	**REC**	35.58	14.64	14.62	10.35	49.25	73.06	90.28	8.08	11.17
18 y	SHAM	SUPINE	**BL**	39.59	13.37	13.35	9.45	55.18	74.82	92.45	6.80	13.60
18 y	SHAM	SUPINE	**REC**	51.23	17.35	17.33	12.27	71.40	78.03	91.81	7.31	12.56
19 y	SHAM	SEATED	**BL**	33.46	20.27	20.24	14.33	45.10	65.06	67.88	30.18	2.25
19 y	SHAM	SEATED	**REC**	32.83	21.38	21.35	15.12	43.90	65.71	67.10	29.84	2.25
19 y	SHAM	SUPINE	**BL**	68.66	59.02	58.94	41.73	87.68	70.47	61.41	32.29	1.90
19 y	SHAM	SUPINE	**REC**	64.07	58.99	58.90	41.71	80.44	70.26	34.46	56.32	0.61
20 Y	GJ	Supine	**BL**	70.0	64.4	64.3	45.5	87.9	77.5	43.8	45.5	1.0
20 Y	GJ	Supine	**REC**	107.3	109.0	108.9	77.1	130.8	74.5	47.3	41.7	1.1
20 Y	GJ	SEATED	**BL**	57.5	87.3	87.2	61.7	52.9	88.7	49.8	41.4	1.2
20 Y	GJ	SEATED	**REC**	67.6	92.8	92.7	65.6	69.5	77.4	23.5	56.4	0.4
21 y	GJ	SEATED	**BL**	106.62	127.09	126.89	89.87	121.07	64.77	36.64	49.72	0.74
21 y	GJ	SEATED	**REC**	132.80	171.96	171.70	121.60	143.14	68.20	42.12	42.17	1.00
21 y	GJ	SUPINE	**BL**	84.57	82.20	82.05	58.12	104.53	58.11	42.63	52.60	0.81
21 y	GJ	SUPINE	**REC**	97.76	106.63	106.43	75.40	115.88	54.99	55.96	41.78	1.34
22 o	GJ	SEATED	**BL**	79.62	82.70	82.58	58.48	96.23	68.86	33.54	55.18	0.61
22 o	GJ	SEATED	**REC**	127.63	122.38	122.20	86.54	158.41	71.53	38.73	44.67	0.87
22 o	GJ	SUPINE	**BL**	63.61	56.61	56.54	40.03	80.55	81.59	41.57	43.34	0.96
22 o	GJ	SUPINE	**REC**	75.36	82.15	82.03	58.09	89.35	72.48	33.72	53.64	0.63
